# Management of Subtrochanteric Proximal Femur Fractures: A Review of Recent Literature

**DOI:** 10.1155/2018/1326701

**Published:** 2018-10-28

**Authors:** Christopher Jackson, Mina Tanios, Nabil Ebraheim

**Affiliations:** Department of Orthopedic Surgery, University of Toledo Medical Center, Toledo, OH 43614-5807, USA

## Abstract

Subtrochanteric femur fractures are an uncommon injury in orthopedics, but when they are encountered they may present difficulties in management. The purpose of this paper is to examine the recent literature on the epidemiology, classification, initial evaluation, and definitely treatment for these injuries. These will assist the physician to determine the optimal treatment strategy and avoid potential surgical complication.

## 1. Introduction

**Figure 1:**
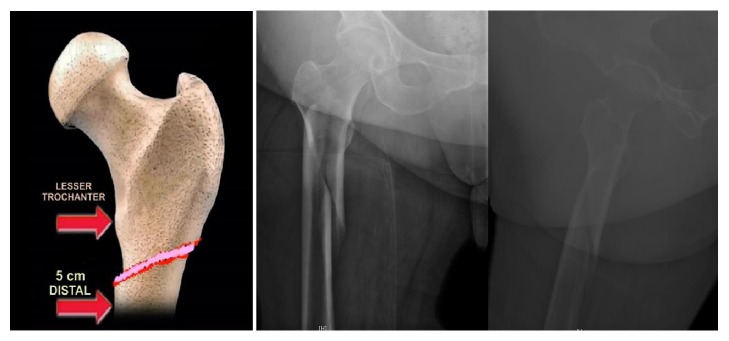
Anatomy of the subtrochanteric area of the femur: the subtrochanteric area of the femur is defined as the area 5 cm distal to lesser trochanter.

Fractures of the proximal femur is a very important topic in the field of orthopedic surgery, but much of that attention is placed on fractures of the femoral neck and intertrochanteric areas. An additional area that must be well understood is the subtrochanteric (ST) region of the femur, which is defined as the proximal femoral shaft located within 5 cm of the lesser trochanter (Figure 1). This area experiences high levels of stress and fractures to this area can result in significant complications and poor clinical outcomes if not managed properly.

## 2. Epidemiology and Risk Factors

The overall incidence rate of fractures of the proximal femur is found to be approximately 230 per 100,000 patients with approximately 5-10% of these fracture occurring within the ST region [1, 2]. Overall the incidence has been estimated to be approximately 15-20/100,000. In terms of age, many studies have found that ST femur fractures occur in a bimodal distribution. One study delineated that approximately two-thirds of all ST fractures occur in patients older than 50 years of age with another 25% occurring in patients aged 17-50 [4]. In terms of gender, females have been found to be at high risk for ST femur fractures with up to a 33% higher incidence rate compared to males [2, 4]. In addition to age and gender, other elicited risk factors include low total bone mineral density, diabetes mellitus, and the use of bisphosphonate medications for the treatment of osteoporosis [5, 6]. The influence of bisphosphonates on the development of atypical femur fractures has garnered significant attention recently with elevated risk with prolongation of therapy, most notably after 5-10 years of treatment [1, 7]. Despite this risk, the overall protection from typical femoral neck and intertrochanteric fractures generated by bisphosphonate use appears to outweigh the increased risk for atypical ST fractures [7]. Overall, due to the prevalence of this injury in the field of orthopedic trauma and with no apparent changes in the incidence in these fractures since 1996 [2] it is prudent for the orthopedic surgeon to have a good understanding of the anatomy of the ST region, presentation, initial work-up, preoperative management, operative stabilization, and postoperative care.

## 3. Anatomy and Biomechanics

**Figure 2:**
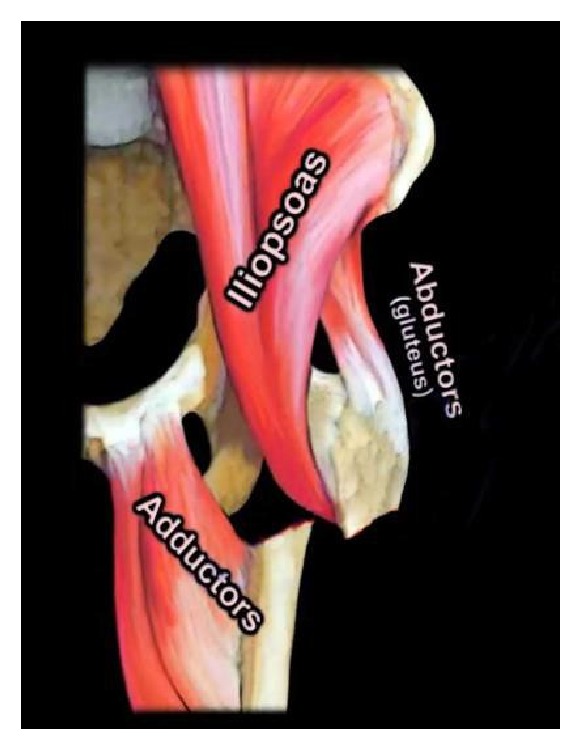
Subtrochanteric fracture deformity: The typical deformity of the subtrochanteric femur fracture is one of external rotation and abduction of the proximal segment and adduction of the distal segment.

As mentioned before the ST area of the proximal femur is defined as the proximal femur that lies 5 cm distal to the lesser trochanter. The primary structural support to this area is the femorale calcar which is a posteromedial structure that begins just distal to the lesser trochanter and travels proximal to support the posteroinferior femoral neck. Early biomechanical studies found that this section of bone can experience up to 1200 N of force within the ST area with standing and gait [8]. These forces are important because while these force can be tolerated in young healthy bone they may overpower weaker osteoporotic bone. In addition to the static forces placed on the proximal femur, this region experiences increased stress secondary to the multiple muscular attachments in the region, which include the lateral hip abductors, medial hip adductors, the iliopsoas, and short external rotators. These muscular attachments have been shown to increase stresses around the hip and proximal femur [9]. In addition to the stresses applied to the ST region, these multiple muscle groups produce predictable deformities patterns that must be understood in order to achieve a proper reduction. The classic deformity that occurs in ST femur fractures is proximal segment abduction, external rotation, and flexion caused by the pull of the gluteus medius, gluteus minimus, the short external rotators, and iliopsoas and adduction of the distal fragment by the gracilis and adductor muscle groups. (Figure 2)

## 4. Presentation: History and Physical Examination

Typically, patients will present in 1 of 2 scenarios. The first one will be a younger patient following a high energy mechanism of injury such as an MVA or fall from height. The patient will usually have multiple injuries and the first priority should be determining whether the patient requires ATLS and volume resuscitation. The second common situation is an elderly patient following a low mechanism of injury which will typically present with an isolated fracture. Following ATLS protocols or in the situation of a low energy MOI, a thorough history should be taken. This should include the MOI, pain, ability to ambulate, the presence of neurologic or vascular symptoms, and the presence of prodromal hip pain or contralateral hip pain. Medication histories should be taken looking for the use of bisphosphonates and the length of therapy.

Examination of the affected lower extremity will reveal a shorted and externally rotated extremity. Injuries are typically closed but examination of the skin should be through as open femur fractures represent extremely serious injuries with significant soft tissue damage. Neurovascular examination should be performed and deficits should be worked up appropriately. Finally, a history of another joint or extremity pain followed by a skeletal survey to rule out other musculoskeletal injuries should be performed.

## 5. Imaging and Classification

**Figure 3:**
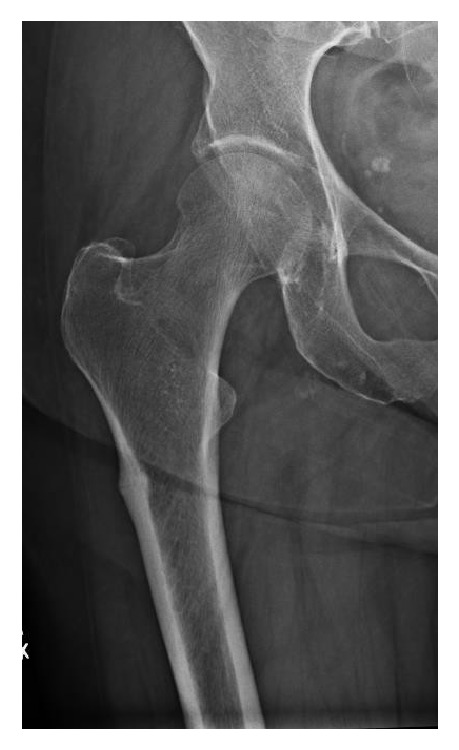
Atypical subtrochanteric femur fracture: Initial identification of atypical subtrochanteric femur fractures can be identified by characteristic lateral cortical thickening.

Initial imaging of the patient with a suspected ST fracture includes an AP pelvis and full-length femur films. These initial imagings will allow for proper injury identification and classification of the fracture. Typical findings will be the proximal fragment resting in abduction, external rotation, and flexion with the distal fragment in adduction. The fracture will also typically be in a long oblique orientation with varying amounts of comminution. In the setting of a history of prolonged bisphosphonate use with or without contralateral hip and thigh pain, the surgeon should attempt to identify atypical fracture patterns associated with bisphosphonates. Recently the American Society for Bone and Mineral Research developed criteria for the identification of atypical ST femur fractures. Common radiographic features of atypical ST femur fractures include transverse fracture patterns with minimal comminution, lateral cortical thinking, and a posteromedial spike in the setting of a low energy injury (Figure 3). The importance of identifying atypical fractures associated with bisphosphonates is the fact that there is a high incidence of bilateral fractures, recommendations for discontinuation of bisphosphonate and conversion to teriparatide, and the need for prophylactic fixation of lateral insufficiency fractures to prevent completion of the fracture [10, 11]. These recommendations come from the overserved improvement in patients with hip pain and lateral cortical thickening with protected weight bearing and conversion from bisphosphonate treatment to teriparatide and lack of improvement with similar treatment in patients with documented radiolucent lines in the lateral cortex. Advanced imaging modalities, such as CT or MRI, can be performed in cases with equivocal plain radiographs to identify occult cortex lucencies. These imaging modalities should also be used to tract the treatment of the stress fractures [3, 10]. Overall several different classification systems have been developed including the Russell-Taylor classification which is based on the presence of lesser trochanter comminution and fracture extension into the piriformis fossa which helped to guide treatment prior to the development of trochanteric start point intramedullary nails. The AO classification system also has a classification system describing fracture morphology and mechanism.

## 6. Emergency Department Management

After performing a history, physical examination, and imaging modalities, initial treatment should always begin with any required resuscitative measures as indicated by ATLS protocols. Following stabilization and ruling out other injuries, the patient may be placed in skeletal traction. Skeletal traction through the use of a distal femoral pin has been shown to restore length of the affected extremity and improve preoperative pain scores [12]. Finally, medical optimization with the assistance of internal medicine/geriatrics has been found to improve inpatient mortality, long-term mortality, and length of stay [13].

## 7. Definitive Management: Nonoperative

Due to the high morbidity and mortality associated with nonoperative management, there are only a few instances where it is acceptable. First the patient in extremis with a high risk of mortality from anesthesia or other medical conditions should avoid surgical treatment. Secondly, hospice patients with minimal discomfort may be treated nonoperatively. However, numerous benefits, such as improved mobility, decrease of pain, and improving the care provided by care givers by the definitive fixation, should be thoroughly discussed with all individuals involved in the medical decision-making process before proceeding with nonoperative management [14].

## 8. Definitive Management: Intramedullary Nailing

**Figure 4:**
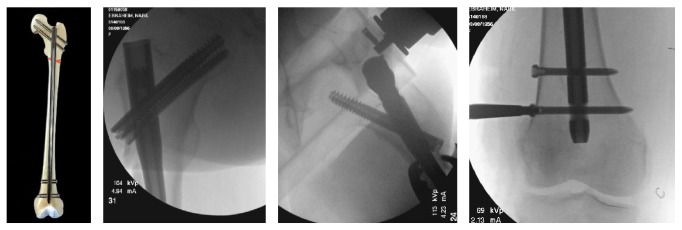
Intramedullary fixation of a subtrochanteric femur fracture: This diagram illustrates and provides radiographs of an example of intramedullary fixation of a subtrochanteric femur fracture. Proximal fixation is achieved through the use of cephalomedullary screws and distally with locked interlock screws.

Overall, the use of intramedullary fixation has become the gold standard for the treatment of ST femur fractures (Figure 4). Overall, intramedullary nailing has been shown to decrease operative time, fixation failure, and hospital length of stay when compared to extramedullary devices [15]. Wiss et al. examined 95 acute ST femur fractures and found average time to union to be 25 weeks with 7 complications including 1 nonunion and 6 malunions [16]. Similar results were found by Shah et al. who examined 51 ST fractures treated with intramedullary nailing and found good results with 1 delayed union in a pathologic fracture secondary to malignancy and 1 case of failure of fixation. This study also revealed overall Harris hip scores of 90.1 at 12 months [16]. Despite the apparent success of intramedullary nailing, slight nuances to the techniques of the procedure have been found to improve outcomes, including the nail starting point, proximal screw orientation, and length of the nail.

In terms of the proximal entry point for an anterograde intramedullary nailing, the surgeon has a choice of a piriformis start point and a greater trochanteric start point. Advantages of the piriformis start point include reduction of the incidence of varus malreduction and medial cortex injury with reaming [17]. Disadvantages are difficulty in obtaining a proper start point in obese patients, patients with hypertrophic short external rotators, or greater trochanter overhang. Additional excessive anterior placement of the starting point by as little as 6 mm can increase the risk of anterior cortical blowout [18]. Finally, there is concern for the proximity of the piriformis start point to the cervical vessels of the medial femoral circumflex artery. The second starting point is the trochanteric start point. Advantages include protecting more of the soft tissue structures around the hip and easier placement [19]. However there is a greater concern for varus malreduction and vast changes in the “ideal” start point based on patient anatomy [3, 20]. Overall the ideal start point must be chosen based on unique patient and fracture characteristics including body habitus, bony anatomy of the proximal femur, and fracture lines into the greater trochanter or basicervical region.

The next technical aspect of anterograde femoral nailing is the proximal screw orientation. For more proximal diaphyseal femoral fractures the crossed proximal screws are used. However this type of construct only provides stabilization through bony contact at the entry site and cortical contact at the interlocking bolt sites. The development of reconstruction nail designs allows for additional fixation via forces between the cephalomedullary screw and the femoral neck and head preventing varus and flexion deformities [21]. In addition to orientation of the screws, the number of screws has been examined. Grisell et al. examined two different two-proximal screw constructs: one with parallel screws into the femoral head and neck and one with crossed screws. This study determined that the axial stiffness was greater in the cross screw construct [22]. In addition, studies have also shown two distal interlock screws provide greater stability than one [21].

Final aspects to intramedullary nailing include nail length, nail size, and nail material. In terms of nail length, the standard of care is full-length intramedullary nails. This is supported by biomechanical studies revealing weak constructs and peri-implant fractures when comparing long and short intramedullary devices [23]. In terms of implant diameter and material, biomechanical studies have shown that larger proximal diameter implants made of stainless steel provided greater fracture stability in rotation, shear, and axial motion over smaller diameter titanium implants [7]. Overall, long intramedullary nailing of ST femur fractures has been shown to provide great outcomes with limited complications.

## 9. Definitive Treatment: Plating

**Figure 5:**
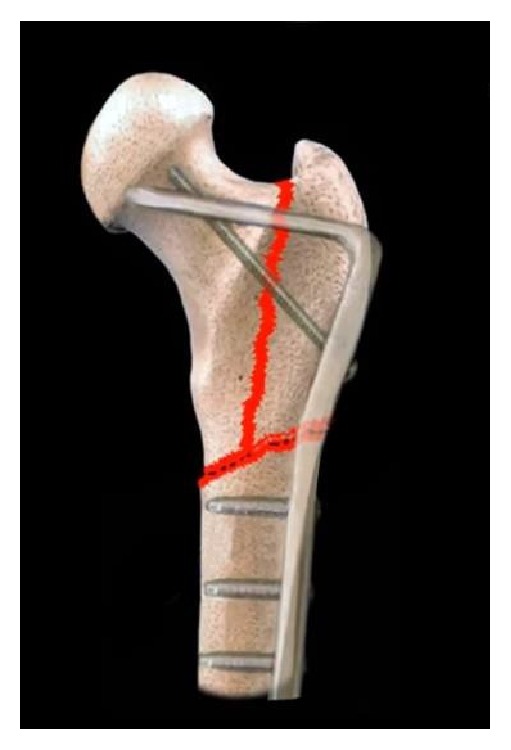
Extramedullary fixation of subtrochanteric femur fractures: This diagram illustrates the use of a 95-degree blade plate construct for the treatment of subtrochanteric femur fractures.

**Figure 6:**
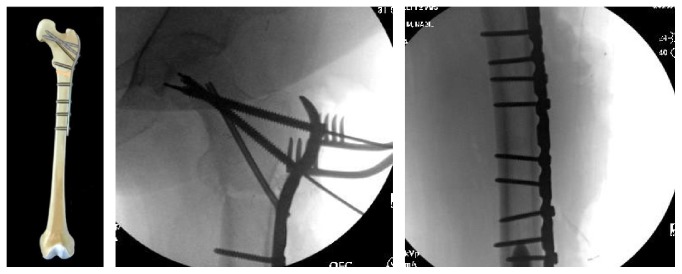
Extramedullary fixation of subtrochanteric femur fractures: This diagram illustrates and provides radiographs of the use of a proximal femoral locking plate construct for the treatment of subtrochanteric femur fractures.

Aside from intramedullary nailing, numerous methods of open reduction and internal fixation of ST femur fractures are available. The most successful type of plating involves the use of 95-degree fixed angle blade plates (Figure 5). Despite some studies revealing moderately good results with fixed angle plating with nonunion rates of approximately 0-10% with times to union of approximately 5 months [2, 24], more recent studies have found less appealing results [25]. These recent changes in outcomes, coupled with the high degree of difficulty in applying this fixed angle devices and decreased infection rates, higher union rates, and shorter operative times with intramedullary nailing, have led to decreased use in 95-degree fixed angle plates [14, 25]. Another form of fixed angle plating includes proximal femoral locking plates (Figure 6). These systems have been shown to have better biomechanical properties than blade plate constructs. However recent studies have also shown poor results including high malunion/nonunion rates and subsequent reoperation rates close to 35% [14, 26]. Finally 135-degree compression plates, commonly used for intertrochanteric hip fractures, have been shown to have failure of fixation upwards of 56% and therefore have been abandoned in the surgical treatment of ST femur fractures [21].

Despite poorer results of open reduction and internal fixation with fixed angle constructs when compared to intramedullary nailing, open plating may still have a prominent role in the acute treatment of ST femur fractures. One study by Robertson et al. showed a large improvement in malunion rates (27% versus 0%) when provisional plating of ST femur fractures was performed prior to intramedullary nailing in fractures that required an open reduction. In addition there was no increase in operative time or blood loss when comparing all cases that required open reduction between the two study groups [15].

## 10. Complications

The complications of surgical treatment of ST femur fractures are similar to the complications for other types of proximal femur fractures, including mortality, nonunion, malunion, and infection. In terms of mortality rates, ST fracture 1-year mortality rates in elderly patients are similar to rates for femoral neck and IT fractures of approximately 9.5% at 30 days, 27% at 1 year, and up to 60% at 4 years [22, 27]. Interestingly, it appears that patients with atypical proximal femur fractures associated with bisphosphonates have a decreased mortality rate at 4 years when compared to typical ST fracture patterns [27].

**Figure 7:**
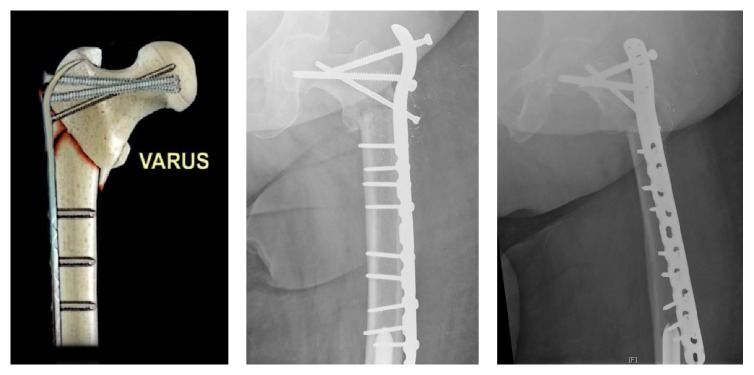
Varus collapse of subtrochanteric femur fractures: This diagram illustrates and provides a radiographic example of a subtrochanteric fracture varus nonunion.

In terms of malunion and nonunion rates, the main cause appears to be inability to obtain anatomic reductions intraoperatively. Development of improper reductions can be the result of improper starting points, especially too lateral with the trochanteric starting point, or lack of direct visualization, instrumentation, or provisional plate fixation prior to intramedullary nail insertion. In addition to increased rates of nonunion, excessive varus and flexion of the proximal segment can cause detrimental changes to gait mechanics. In general, the typical failure will result in a varus deformity (Figure 7) and these complications are typically treated with exchange intramedullary nailing or fixed angle plate constructs with or without bone grafting [14].

In terms of infection, both deep and superficial surgical site infections can occur following operative treatment of ST femur fractures. Typically superficial soft tissue infections can be treated conservatively with antibiotic alone. In terms of deep infections, they may be approached with surgical irrigation and debridement with antibiotics in an attempt to allow the fracture to heal prior to implant removal. However, should the patient be found to have a persistent infected nonunion, removal of the implant is needed followed by temporary fixation with an antibiotic intramedullary device followed by antibiotics until the infection has been effectively treated [14].

## 11. Summary

ST femur fractures are a less common type of hip fracture but can occur in both young and elderly patients after both high- and low-energy mechanisms of injury. While initial evaluation and treatment involve resuscitation and modalities such as skeletal traction, very rarely should treatment not proceed to surgical fixation. In terms of fixation, intramedullary nailing is the gold standard of treatment and can be performed safely for both typical and atypical ST fractures. However there are many technical aspects to intramedullary nailing that a surgeon must consider. While fixed angle constructs can be used for initial treatment of fixation, these methods are more commonly utilized for ST femoral malunion/nonunion treatment.
